# The Role of p16/Ki-67 Immunostaining, hTERC Amplification and Fibronectin in Predicting Cervical Cancer Progression: A Systematic Review

**DOI:** 10.3390/biology11070956

**Published:** 2022-06-23

**Authors:** Septimiu Toader Voidăzan, Caterina Dianzani, Mădălina Aurelia Husariu, Bíborka Geréd, Sabin Gligore Turdean, Cosmina Cristina Uzun, Zsolt Kovacs, Florin Francisc Rozsnyai, Nicoleta Neagu

**Affiliations:** 1 Department of Epidemiology, George Emil Palade University of Medicine, Pharmacy, Science and Technology of Târgu Mureş, 540139 Târgu Mureş, Romania; septimiu.voidazan@umfst.ro; 2 Plastic and Reconstructive Surgery Unit, Campus Biomedico University of Rome, 00128 Rome, Italy; c.dianzani@unicampus.it; 3 Dermatology Clinic, Mureș County Hospital, 540136 Târgu Mureș, Romania; husariumadalina@gmail.com (M.A.H.); geredbiborka@gmail.com (B.G.); 4 Department of Pathology, George Emil Palade University of Medicine, Pharmacy, Science and Technology of Târgu Mureş, 540139 Târgu Mureș, Romania; sabin.turdean@umfst.ro; 5 Department of Biochemistry, Environmental Chemistry, George Emil Palade University of Medicine, Pharmacy, Science and Technology of Târgu Mureş, 540139 Târgu Mureș, Romania; cosmina20uzun@gmail.com (C.C.U.); zsolt.kovacs@umfst.ro (Z.K.); 6 Department of Obstetrics Gynecology, George Emil Palade University of Medicine, Pharmacy, Science and Technology of Târgu Mureş, 540139 Târgu Mureș, Romania; florin.rozsnyai@umfst.ro

**Keywords:** cervical cancer, HPV, p16, Ki-67, telomerase, fibronectin, progression

## Abstract

**Simple Summary:**

Human papillomaviruses (HPV) are common sexually transmitted infections and they are responsible for cervical cancer (CC), as well as for several other anogenital cancers. CC is the fourth leading cause of death in women with cancer, although it could be preventable by enforcement of optimal screening programs. The Pap smear is the standard screening test for CC and precancerous lesions, and a combination of Pap smear and HPV testing is generally recommended as a triage step before colposcopy. However, these tests cannot predict lesion progression, which is why several adjunctive biomarkers have been studied. Our aim was to summarize current scientific data on the role of these biomarkers, with a view to determining which biomarkers could help to more accurately establish the need for colposcopy and at the same time, to limit the number of unnecessary colposcopy referrals.

**Abstract:**

Human papillomaviruses (HPVs) are common sexually transmitted infectious agents responsible for several anogenital and head and neck cancers. Cervical cancer (CC) is the fourth leading cause of death in women with cancer. The progression of a persistent HPV infection to cancer takes 15–20 years and can be preventable through screening. Cervical cytology (Pap smear) is the standard screening test for CC and precancerous lesions. For ASC-US and ASC-H lesions, a combination of Pap smear and HR-HPV analysis is recommended as a triage step before colposcopy. However, these tests cannot predict progression to CC. For this purpose, we summarized current scientific data on the role of p16/Ki-67 immunohistostaining, telomerase and fibronectin in predicting progression to CC. p16 and p16/Ki-67 dual staining (DS) were more specific than HR-HPV DNA testing for the detection of CIN2+/CIN3+ in women with ASC-US and LSIL. Similarly, hTERC FISH analysis significantly improved the specificity and positive predictive value of HPV DNA testing in differentiating CIN2+ from CIN2 cytological samples. In conclusion, p16 IHC, p16/Ki-67 DS and hTERC FISH amplification are all valid adjunctive biomarkers which significantly increase the sensitivity and specificity of cervical dysplasia diagnosis, especially when combined with HPV DNA testing. However, considering the global socioeconomic background, we can postulate that p16 and p16/ Ki-67 IHC can be used as a next step after positive cytology for ASC-US or LSIL specimens in low-income countries, instead of HPV DNA testing. Alternatively, if HPV DNA testing is covered by insurance, p16 or p16/Ki-67 DS and HPV DNA co-testing can be performed. In middle- and high-income countries, hTERC amplification can be performed as an adjunctive test to HPV DNA testing in women with ASC-US and LSIL.

## 1. Introduction

Human papillomaviruses (HPVs) are common sexually transmitted infectious agents described as non-enveloped, double-stranded, circular DNA viruses belonging to the Papovaviridae family [1]. Approximately 90% of HPV infections are transient and become undetectable in 1–2 years. However, persistent infections with oncogenic HPV types have been associated with the progression of the disease [2,3]. According to epidemiological data, 12 mucosal alpha HPVs are categorized as high-risk HPV (HR-HPV) types and are responsible for several anogenital and head and neck cancers [4]. HPV16 and 18 are the most carcinogenic types: HPV16 has been associated with 50–60% of cervical cancers (CCs), HPV18 with 10–15% of CCs and the remaining HR-HPV types have been implicated in 25–40% of CCs [5,6].

CC is the fourth most frequently diagnosed cancer worldwide [7] and according to the WHO it is the fourth leading cause of death in women with cancer, with an estimated annual incidence of 604,000 cases and 342,000 deaths reported worldwide in 2020 [8]. The progression of a persistent HPV infection to cancer usually takes 15–20 years and it is preventable by the optimal application of secondary prevention programs [9]. CC screening is recommended to be initiated at the age of 21 years via cytology every three years or, for women aged 30–65 years, cytology in combination with HR-HPV testing every five years. Screening can be discontinued in women with a hysterectomy or women older than 65 years who have a history of regular screening with negative results [10,11,12].

Cytology-based screening, also known as the Papanicolau smear (Pap smear) test, was first introduced in 1940 by Georgios Papanicolau as a CC screening tool. Conventionally, microscopic evaluation is performed on cervical cells obtained from cervical scraping after fixing them on a glass slide. Another cytology-based screening method is liquid-based cytology (LBC), by which cervical cells are suspended in a liquid medium and then filtered and transferred onto a monolayer for microscopic evaluation [11,13,14]. Both methods have shown similar sensitivity, specificity, positive predictive value, negative predictive value and accuracy for the detection of cervical intraepithelial neoplasia (CIN) 2 or higher [15].

Cytological findings have been classified according to the Bethesda system [16], which was updated in 2014 [17] and includes the following categories: atypical squamous cells of undetermined significance (ASC-US); atypical squamous cells, cannot exclude high-grade squamous intraepithelial lesion (ASC-H); low-grade squamous intraepithelial lesion (LSIL—corresponding to mild dysplasia/ CIN 1); high-grade squamous intraepithelial lesion (HSIL—corresponding to moderate or severe dysplasia, CIS; CIN 2 or CIN 3) and squamous cell carcinoma (SCC) [17]. ASC-US and LSIL are generally considered transient lesions of the cervical epithelium, although an important proportion of women with ASC-US and LSIL have underlying CIN2 or 3 and an increased risk for developing CC [18]. Rigurous triage of women with ASCUS or LSIL is warranted for early diagnosis and treatment of CIN2 or 3 lesions, as well as for minimizing unnecessary biopsies, especially in young women who wish to conceive. The Pap smear test is currently used as a first step in the CC screening method and more recently, HR-HPV co-testing has been integrated into cervical cancer screening guidelines [19]. Despite existing protocols, CC maintains high incidence and mortality rates, which is why several adjunctive biomarkers and their role in accurately predicting progression to CC have been studied. For this purpose, we have summarized current scientific data on the role of p16/Ki-67 immunohistostaining (IHC), telomerase and fibronectin biomarkers. 

The main role of p16/Ki-67 IHC in the triage of HPV-positive women is to distinguish between those with underlying high- and low-grade cervical lesions, which aids in determining the necessity for immediate colposcopy referrals [20]. It is cost-effective, highly reproducible and has a relatively low technical complexity [13], which makes it easily accessible and widely used.

Telomerase up-regulation is known to arrest cellular apoptosis, thus having a central role in malignant proliferation [21,22]. Moreover, the E6/E7 oncogene encoding the HPV proto-oncoprotein can up-regulate telomerase activity by human telomerase RNA component (hTERC) gene amplification. Studies have shown an important correlation between HR-HPV infection and hTERC up-regulation in CC progression [23,24,25,26]. Telomerase activity as a prognostic biomarker in CC has been demonstrated through numerous studies and it is generally recommended as an ancillary biomarker in CC screening, after cytology and HPV DNA detection.

Fibronectin (FN1) is a glycoprotein component of the extracellular matrix that plays an important part in cell growth, cell adhesion and differentiation [27]. A few studies have discussed its potential role in different malignancies such as hepatocellular, renal, gastrointestinal, head and neck cancers [28,29]. We further discuss the literature published so far.

## 2. Materials and Methods

### 2.1. Study Selection

We conducted a systematic review of the literature following the Preferred Reporting Items for Systematic Reviews and Meta-Analyses (PRISMA) guidelines. We searched the PubMed database for studies published between 2011 and 2022 using the term *cervical cancer* in combination with the following terms: *telomerase*, *fibronectin*, *p16*, *ki-67*, *HPV.* The last search was run on 25th March 2022. There was no limit to study design.

### 2.2. Data Extraction

Two investigators independently selected relevant articles according to predefined inclusion and exclusion criteria, as described above. Disagreements were resolved by discussion, with a prior arrangement that any unsettled discrepancy would be determined by a third author.

### 2.3. Inclusion Criteria

Eligibility was restricted to studies in which p16, ki-67, telomerase and fibronectin positivity were correlated with histopathologic modifications in cervical specimens classified according to the Bethesda system. The relationship between the grade of cervical dysplasia and mentioned markers was analyzed. Only articles in English were selected. Only studies in which telomerase activity and HPV detection were performed by genomic amplification techniques and not by staining procedures were included. Other potentially relevant articles were identified by manually checking the references of the articles included.

### 2.4. Exclusion Criteria

We excluded the following studies: those where the number of patients was either not specified or expressed as different age frequencies; those where the main inclusion criterion was only HPV-positive patients; those where different comparisons were drawn, either between the sensitivity and specificity of various detection methods, or between self-sampled specimens and samples collected by healthcare professionals. Additionally, studies in which p16 and ki-67 staining were assessed only according to the level of expression and not as either positive or negative specimens were excluded, given the great heterogeneity of histopathological assessment techniques and grading systems [30,31].

### 2.5. Data Synthesis and Statistical Analysis

Pertinent data were selected in the form of: number of biopsy specimens analyzed, number of specimens for each category of the Bethesda classification system, number of HPV-positive specimens, HPV types detected, number of p16-, Ki-67-, telomerase- and fibronectin-positive specimens.

### 2.6. Limitations

The limitations of this review lie in study heterogeneity, which is reflected in the different scoring systems used for cervical modifications, for HPV detection, and for p16/ ki-67 staining positivity. In order to limit bias in reporting, we objectively summarized relevant data from the literature in Tables 1–4. We only included studies where a definitive histopathologic diagnosis was provided and cervical dysplasia was classified according to either of the Bethesda systems [16,17]. Non-neoplastic lesions (NNL) included any type of modification, including inflammation (cervicitis), infection and atypical metaplasia, which was mentioned in just one study [32]. Cervical cancer (CC) was ascribed to both squamous cell carcinoma, either in situ or invasive, and adenocarcinoma, considering that most studies included both types of cervical cancer under this common nomenclature.

## 3. Results

**Figure 1:**
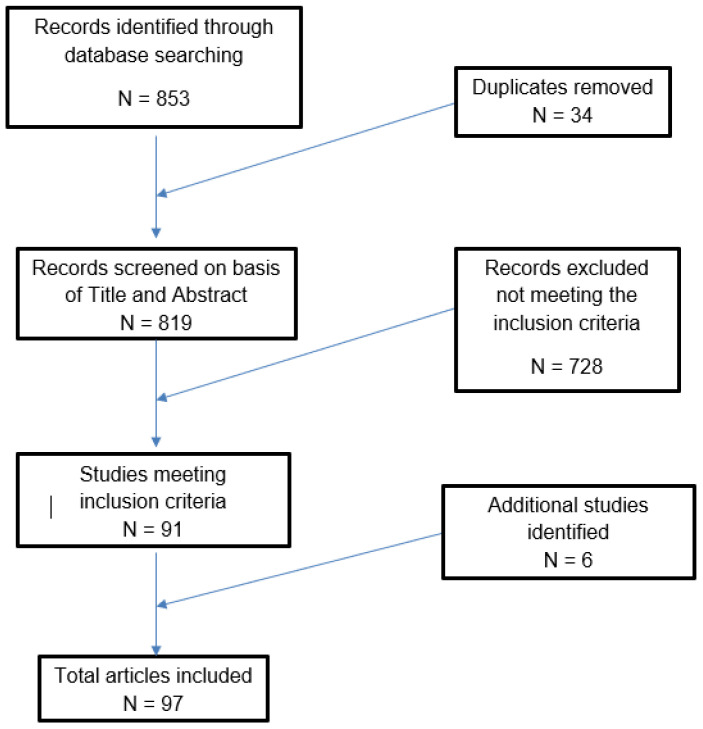
Literature search and article selection.

A total of 853 records were initially identified in the literature search, of which 34 were duplicates and 728 did not meet the inclusion criteria, thus being further excluded (Figure 1). A total of 20,877 biopsy specimens were investigated, of which there were 7174 for p16 IHC, 745 for Ki-67 IHC, 5329 for p16/ ki-67 dual staining (DS) and 9084 for telomerase up-regulation.

**p16 staining**

Nineteen studies had relevant data regarding p16 IHC, totaling 7174 biopsy specimens: 1375 NNL, 1857 CIN1, 2 CIN 1/2, 1923 CIN2, 43 CIN2/3, 1664 CIN3, 310 CC. p16 was positive in 3813/7069 biopsy specimens: 2.54% NNL, 15.02% CIN1, 0.05% CIN1/2, 35.79% CIN2, 0.91% CIN2/3, 38.18% CIN3, 6.74% CC. HPV genotyping was positive in 4486/6335 biopsy specimens: 5.84% NNL, 19.30% CIN1, 0.02% CIN1/2, 35.95% CIN2, 0.53% CIN2/3, 32.56% CIN3, 4.34% CC (Table 1).

The majority of the studies demonstrated a directly proportional increase in the likelihood of p16 positive staining and the severity of cervical dysplasia [6,33,34,35,36,37,38,39,40,41,42,43]. Similarly, the number of HPV-positive specimens increased with the degree of intraepithelial lesion [6,33,34,35,36,37,43]. Moreover, higher sensitivity and specificity rates were demonstrated for the combination of HR-HPV detection and p16 IHC in the early diagnosis of cervical lesions, as compared with either test alone: p16 sensitivity (Se) = 95.83% and specificity (SP) = 65.34%, HR-HPV Se = 91.67% and Sp = 53.4%, combination p16 and HR-HPV testing Se = 89.58% and Sp = 72.73% [33]. 

Additionally, Alhamlan et al. [44] found that p16 IHC was a significant negative predictor of survival. In a retrospective, cross-sectional study conducted on 315 cervical biopsy specimens collected from women aged 23–95 years old who were also PCR-tested for HPV L1 protein, p16 overexpression correlated with poorer survival rates (multivariate Cox regression, hazard ratio, 3.2; 95% CI, 1.1–8.8). Conversely, a multivariate Cox regression analysis showed that HPV-positive cervical cancer (CC) had better survival rates, whereas HPV-negative CC was linked to significantly worse disease-free survival [36]. Similar findings were reported in the literature [45,46,47].

**Table 1:** p16 IHC in cervical tissue biopsy specimens.

Reference, Year	Number of Biopsy Specimens	HPV Detection and Correlation with Biopsy Results	p16 Positive IHC and Correlation with Biopsy Results
Alhamlan et al., 2021 [44]	315, of which:	96/315, of which:	111/212, of which:
	82 NNL	6/82 NNL	2/54 NNL
	54 CIN1	6/54 CIN1	9/37 CIN1
	16 CIN2	3/16 CIN2	7/10 CIN2
	45 CIN3	17/45 CIN3	20/25 CIN3
	118 CC	64/118 CC	73/84 CC
Castle et al., 2019 [6]	4010, of which: 283 NNL	3172/4010	2520/4010, of which:
	934 CIN1	59/283 NNL	21/283 NNL
	1512 CIN2	507/934 CIN1	248/934 CIN1
	1208 CIN3	1386/1512 CIN2	1087/1512 CIN2
	73 CC	1154/1208 CIN3	1095/1208 CIN3
		66/73 CC, of which:	69/73 CC
		1283/3172 HPV16:	
		9/283 NNL	
		67/934 CIN1	
		506/1512 CIN2	
		658/1208 CIN3	
		43/73 CC	
		242/3172 HPV 18/45:	
		7/283 NNL	
		49/934 CIN1	
		111/1512 CIN2	
		65/1208 CIN3	
		10/73 CC	
		1357/3172 OHR-HPV:	
		28/283 NNL	
		270/934 CIN1	
		659/1512 CIN2	
		390/1208 CIN3	
		10/73 CC	
		213/3172 IR-HPV:	
		11/283 NNL	
		82/934 CIN1	
		85/1512 CIN2	
		34/1208 CIN3	
		2/73 CC	
		76/3172 LR-HPV:	
		4/283 NNL	
		39/934 CIN1	
		25/1512 CIN2	
		7/1208 CIN3	
		1/73 CC	
Haltas et al., 2012 [48]	64, of which:	N/A	37/64, of which:
	8 NNL		0/8 NNL
	26 CIN1		12/26 CIN1
	19 CIN2		15/19 CIN2
	8 CIN3		7/8 CIN3
	3 CC		3/3 CC
Huang et al., 2011 [33]	272, of which:	170/272, of which: (HR-HPV)	153/272, of which:
	82 NNL	19/82 NNL	14/82 NNL
	94 CIN1	63/94 CIN1	47/94 CIN1
	41 CIN2	35/41 CIN2	37/41 CIN2
	28 CIN3	27/28 CIN3	28/28 CIN3
	27 CC	26/27 CC	27/27 CC
Indarti et al., 2013 [34]	30, of which:	14/30, of which:	17/30
	11 CIN1	0/11 CIN1	0/11 CIN1
	9 CIN2	5/9 CIN2	7/9 CIN2
	10 CIN3	9/10 CIN3	10/10 CIN3
Liao et al., 2013 [35]	463, of which:	248/463	160/463
	187 NNL	29/187 NNL	5/187 NNL
	171 CIN1	124/171 CIN1	73/171 CIN1
	53 CIN2	45/53 CIN2	40/53 CIN2
	49 CIN3	47/43 CIN3	39/49 CIN3
	3 CC	3/3 CC	3/3 CC
Ma et al., 2011 [36]	131, of which:	88/131 HR-HPV, of which:	49/131
	79 NNL	43/79 NNL	10/79 NNL
	26 CIN1	21/26 CIN1	16/26 CIN1
	23 CIN2/3	21/23 CIN2/3	20/23 CIN2/3
	3 CC	3/3 CC	3/3 CC
Pabuccu et al., 2017 [49]	27, of which:	N/A	13/27
	14 NNL		1/14 NNL
	5 CIN1		5/5 CIN1
	8 CIN2/3		7/8 CIN2/3
Pacchiarotti et al., 2014 [50]	577, of which:	N/A	193/577, of which:
	312 NNL		6/312 NNL
	159 CIN1		91/159 CIN1
	39 CIN2		36/39 CIN2
	58 CIN3		53/58 CIN3
	9 CC		7/9 CC
Sarma et al., 2017 [51]	110, of which:	N/A	60/110, of which:
	25 NNL		2/25 NNL
	25 CIN1		8/25 CIN1
	21 CIN2		11/21 CIN2
	12 CIN3		12/12 CIN3
	27 CC		27/27 CC
Tsoumpou et al., 2011 [52]	126, of which:	64/126, of which:	28/126, of which:
	12 NNL	28/78 NNL/CIN1	8/78 NNL/CIN1
	66 CIN1	36/48 CIN2/3	20/48 CIN2/3
	36 CIN2		
	12 CIN3		
Valasoulis et al., 2013 [37]	200, of which:	133/200 HPV:	53/200, of which:
	23 NNL	6/23 NNL	2/23 NNL
	79 CIN1	41/79 CIN1	12/79 CIN1
	50 CIN2	41/50 CIN2	17/50 CIN2
	48 CIN3	45/48 CIN3	22/48 CIN3
		118/200 HR-HPV:	
		5/23 NNL	
		30/79 CIN1	
		38/50 CIN2	
		45/48 CIN3	
		60/200 HPV16/18:	
		0/23 NNL	
		14/79 CIN1	
		17/50 CIN2	
		29/48 CIN3	
van Baars et al., 2015 [39]	104, of which:	90/104, of which:	76/104, of which:
	25 NNL	13/25 NNL	0/25 NNL
	11 CIN1	11/11 CIN1	8/11 CIN1
	23 CIN2	23/23 CIN2	23/23 CIN2
	45 CIN3	43/45 CIN3	45/45 CIN3

**p16/Ki-67 DS**

Seventeen studies had relevant data regarding p16/Ki-67 DS, totaling 5329 biopsy specimens: 2704 NNL, 936 CIN1, 2 CIN1/2, 655 CIN2, 12 CIN2/3, 810 CIN3, 210 CC. p16/Ki-67 DS was positive in 2327/5300 biopsy specimens: 20.24% NNL, 15.68% CIN1, 22.04% CIN2, 0.34% CIN2/3, 30.46% CIN3, 8.51% CC. HPV genotyping was positive in 2376/4883 biopsy specimens: 28.78% NNL, 17.97% CIN1, 0.04% CIN1/2, 18.01% CIN2, 0.37% CIN2/3, 24.41% CIN3, 7.53% CC.

p16/Ki-67 IHC has been used most frequently throughout the studies. Similar to p16 and Ki-67 IHC alone, an increase in the number of DS-positive biopsy specimens was correlated with a more severe histological diagnosis [32,41,42,43,53,54,55,56,57,58,59,60,61,62,63] and with HPV DNA positivity [41,42,43,58,59,63] (Table 2).

**Table 2:** p16/Ki-67 DS in cervical tissue biopsy specimens.

Reference, Year	Number of Biopsy Specimens	HPV Detection and Correlation with Biopsy Results	p16/Ki67 Positive IHC and Correlation with Biopsy Results
Celewicz et al., 2018 [53]	43, of which:	NA	30/43, of which:
	17 NNL		9/17 NNL
	5 CIN1		2/5 CIN1
	10 CIN2		9/10 CIN2
	8 CIN3		7/8 CIN3
	3 CC		3/3 CC
Diouf et al., 2020 [54]	69, of which:	30/38, of which:	32/46, of which:
	30 NNL	1/7 NNL	1/7 NNL
	14 CIN1	4/6 CIN1	6/14 CIN1
	3 CIN2	6/6 CIN2/3	6/6 CIN2/3
	3 CIN3	19/19 CC	19/19 CC
	19 CC		
Donà et al., 2012 [64]	113, of which:	95/107	62/107, of which:
	14 NNL	5/13 NNL	0/13 NNL
	35 CIN1	31/33 CIN1	13/33 CIN1
	24 CIN2	23/24 CIN2	17/24 CIN2
	37 CIN3	36/37 CIN3/CC	32/37 CIN3/CC
	3 CC		
		84/107 HR-HPV	
		3/13 NNL	
		25/33 CIN1	
		20/24 CIN2	
		36/37 CIN3/CC	
		11/107 O-HPV	
		2/13 NNL	
		6/33 CIN1	
		3/24 CIN2	
		0/37 CIN3/CC	
El-Zein et al., 2020 [55]	492, of which:	321/492, of which:	279/492, of which:
	134 NNL	47/134 NNL	41/134 NNL
	130 CIN1	69/130 CIN1	54/130 CIN1
	99 CIN2	86/99 CIN2	72/99 CIN2
	121 CIN3	111/121 CIN3	105/121 CIN3
	8 CC	8/8 CC	7/8 CC
		119/492 HPV16:	
		7/134 NNL	
		17/130 CIN	
		37/99 CIN2	
		55/121 CIN3	
		3/8 CC	
		26/492 HPV18:	
		6/134 NNL	
		4/130 CIN1	
		5/99 CIN2	
		5/121 CIN3	
		6/8 CC	
		139/492 HPV16/18:	
		12/134 NNL	
		20/130 CIN1	
		41/99 CIN2	
		58/121 CIN3	
		8/8 CC	
		235/492 OHR-HPV:	
		41/134 NNL	
		63/130 CIN1	
		58/99 CIN2	
		70/121 CIN3	
		3/8 CC	
		321/492 ANY HR-HPV:	
		47/134 NNL	
		69/130 CIN1	
		86/99 CIN2	
		111/121 CIN3	
		8/8 CC	
Frega et al., 2019 [56]	78, of which:	73/78, of which:	74/78, of which:
	53 CIN2	50/53 CIN2	50/53 CIN2
	25 CIN3	23/25 CIN3	24/25 CIN3
Liu et al., 2020 [65]	305, of which:	N/A	165/305, of which:
	90 NNL		3/90 NNL
	48 CIN1		8/48 CIN1
	35 CIN2		26/35 CIN2
	117 CIN3		113/117 CIN3
	15 ICC		15/15 CC
Ngugi et al., 2015 [57]	22, of which:	21/22 HR-HPV, of which:	8/22, of which:
	12 NNL	11/12 NNL	1/12 NNL
	2 CIN1	2/2 CIN1	0/2 CIN1
	2 CIN2	2/2 CIN2	1/2 CIN2
	6 CIN3	6/6 CIN3	6/6 CIN3
Waldstrøm et al., 2013 [59]	226, of which:	174/226, of which:	154/226, of which:
	42 NNL	28/42 NNL	23/42 NNL
	97 CIN1	66/97 CIN1	54/97 CIN1
	41 CIN2	36/41 CIN2	33/41 CIN2
	45 CIN3	43/45 CIN3	43/45 CIN3
	1 CC	1/1 CC	1/1 CC
Wentzensen et al., 2012 [32]	623, of which:	171/623 HPV16, of which:	371/623, of which:
	137 NNL	24/137 NNL	42/137 NNL
	228 CIN1	31/228 CIN1	106/228 CIN1
	169 CIN2	60/169 CIN2	140/169 CIN2
	83 CIN3	53/83 CIN3	77/83 CIN3
	6 CC	3/6 CC	6/6 CC
Yu et al., 2016 [60]	1290, of which:	463/1290, of which:	427/1290, of which:
	996 NNL	204/996 NNL	183/996 NNL
	63 CIN1	41/63 CIN1	34/63 CIN1
	42 CIN2	40/42 CIN2	34/42 CIN2
	119 CIN3	111/119 CIN3	111/119 CIN3
	70 CC	67/70 CC	65/70 CC
Yu et al., 2016 [61]	701, of which:	173/701, of which:	149/701, of which:
	640 NNL	126/640 NNL	111/640 NNL
	46 CIN1	32/46 CIN1	26/46 CIN1
	11 CIN2	11/11 CIN2	8/11 CIN2
	4 CIN3	4/4 CIN3	4/4 CIN3
Zhang et al., 2019 [62]	537, of which:	294/537, of which:	234/537, of which:
	298 NNL	76/298 NNL	39/298 NNL
	29 CIN	18/29 CIN	10/29 CIN
	49 CIN2	45/49 CIN2	38/49 CIN2
	111 CIN3	106/111 CIN3	99/111 CIN3
	50 CC	49/50 CC	48/50 CC
		168/537 HPV16/18	
		23/298 NNL	
		8/29 CIN	
		16/49 CIN2	
		80/111 CIN3	
		41/50 CC	
		168/537 O-HPV	
		59/298 NNL	
		10/29 CIN	
		34/49 CIN2	
		50/111 CIN3	
		15/50 CC	
Zhu et al., 2019 [63]	300, of which:	256/300, of which:	96/300, of which:
	138 NNL	103/138 NILM	3/138 NILM
	108 CIN1	100/108 CIN1	40/108 CIN1
	29 CIN2	28/29 CIN2	28/29 CIN2
	22 CIN3	22/22 CIN3	22/22 CIN3
	3 CC	3/3 CC	3/3 CC

**Ki-67 staining**

Six studies had data regarding Ki-67 IHC alone, totaling 745 biopsy specimens, of which: 243 NNL, 196 CIN1, 2 CIN1/2, 104 CIN2, 12 CIN2/3, 141 CIN3, 47 CC. Ki-67 was positive in 384/654 biopsy specimens: 17.18% NNL, 18.48% CIN1, 0.26% CIN1/2, 22.39% CIN2, 2.60% CIN2/3, 27.60% CIN3, 13.54% CC. HPV genotyping was positive in 411/684 specimens: 21.16% NNL, 22.62% CIN1, 0.24% CIN1/2, 18.24% CIN2, 0.73% CIN2/3, 28.95% CIN3, 8.02% CC.

Ki-67 was generally expressed in combination with p16 IHC, as DS positivity. Where data were available, a direct proportionality relation between Ki-67 expression alone and the severity of intraepithelial lesion was demonstrated [40,41,42,43,66], as well as between Ki-67 expression and HPV DNA positivity [40,41,42,43] (Table 3).

**Table 3:** p16, Ki-67 and DS IHC in cervical tissue biopsy specimens.

Reference Year	Number of Biopsy Specimens	HPV Detection and Correlation with Biopsy Results	p16 Positive IHC and Correlation with Biopsy Results	KI-67 Positive IHC	DS Positive IHC and Correlation with Biopsy Results
				and Correlation with Biopsy Results	
Chang et al., 2014 [40]	143, of which:	70/143, of which:	31/141, of which:	29/124 of which:	NA
	77 NNL	23/77 NNL	5/75 NNL	2 /69 NNL	
	33 CIN	21/33 CIN1	3/33 CIN1	2/27 CIN1	
	6 CIN2	4/6 CIN2	4/6 CIN2	4/5 CIN2	
	22 CIN3	18/22 CIN3	15/21 CIN3	17/19 CIN3	
	5 CC	4/5 CC	4/5 CC	4/4 CC	
Gatta et al., 2011 [67]	72, of which:	9/72, of which:	41/72, of which:	N/A	NA
	10 NNL (controls)	0/10 NNL	0/10 NNL		
	32 CIN1	8/32 CIN1	11/32 CIN1		
	10 CIN2	1/10 CIN2	10/10 CIN2		
	10 CIN3	0/10 CIN3	10/10 CIN3		
	10 CC	0/10 CC	10/10 CC		
Jackson et al., 2012 [43]	97, of which:	17/36, of which:	14/97, of which:	25//97, of which:	13/97, of which:
	39 NNL	4/9 NNL	1/39 NNL1	4/39 NNL	1/39 NNL
	46 CIN1	10/24 CIN1	5/46 CIN1	11/46 CIN1	4/46 CIN1
	12 CIN2/3	3/3 CIN2/3	8/12 CIN2/3	10/12 CIN2/3	8/12 CIN2/3
Koo et al., 2013 [41]	70, of which:	36/70 HR-HPV:	50/70, of which:	48/70, of which:	43/70, of which:
	27 NNL	9/27 NNL	15/27 NNL	16/27 NNL	4/27 NNL
	6 CIN1	2/6 CIN1	2/6 CIN1	2/6 CIN1	4/6 CIN1
	20 CIN2	14/20 CIN2	16/20 CIN2	14/20 CIN2	18/20 CIN2
	17 CIN3	11/17 CIN3	17/17 CIN3	16/17 CIN3	17/17 CIN3
		of which:			
		18/36 HPV 16/18:			
		3/9 NNL			
		0/2 CIN1			
		7/14 CIN2			
		8/11 CIN3			
Li et al., 2019 [42]	350, of which:	271/350, of which:	197/350, of which:	276/350, of which:	185/350
	84 NNL	49/84 NNL			
	77 CIN1	50/77 CIN1	9/84 NNL	41/84 NNL	8/84 NNL
	68 CIN2	56/68 CIN2	22/77 CIN1	56/77 CIN1	17/77 CIN1
	89 CIN3	87/89 CIN3	55/68 CIN2	60/68 CIN2	50/68 CIN2
	32 CC	29/32 CC	80/89 CIN3	87/89 CIN3	79/89 CIN3
			31/32 CC	32/32 CC	31/32 CC
		271/350, of which:			
		141/350 HPV16			
		16/350 HPV 18			
		16/350 HPV 31			
		21/350 HPV 33			
		13/350 HPV 35			
		13/350 HPV 39			
		3/350 HPV 45			
		16/350 HPV 51			
		56/350 HPV 52			
		10/350 HPV 56			
		61/350 HPV 58			
		8/350 HPV 59			
		11/350 HPV 68			
Toll et al., 2014 [66]	13, of which:	8/13, of which:	10/13, of which:	6/13, of which:	5/13, of which:
	6 NNL	2/6 NNL	4/6 NNL	3/6 NNL	2/6 NNL
	2 CIN1	2/2 CIN1	1/2 CIN1	0/2 CIN1	0/2 CIN1
	2 CIN1/2	1/2 CIN1/2	2/2 CIN1/2	1/2 CIN1/2	1/2 CIN1/2
	3 CIN3	3/3 CIN3	3/3 CIN3	2/3 CIN3	2/3 CIN3

**Telomerase**

Seventeen studies had data regarding telomerase up-regulation detected via fluorescence in situ hybridization (FISH) of hTERC amplification, totaling 9084 biopsy specimens: 1998 NNL, 2423 CIN1, 65 CIN1/2, 1617 CIN2, 120 CIN2/3, 1832 CIN3, 1029 CC. Telomerase was detected in 4337/9084 biopsy specimens: 4.28% NNL, 12.12% CIN1, 0.94% CIN1/2, 24.53% CIN2, 1.86% CIN2/3, 34.67% CIN3, 22.09% CC. HPV genotyping was positive in 2872/ 3937 biopsy specimens: 12.74% NNL, 23.39% CIN1, 1.11% CIN1/2, 17.79% CIN2, 2.09% CIN2/3, 25.52% CIN3, 11.90% CC (Table 4).

Throughout the studies, telomerase activity increased with the severity of cervical dysplasia [67,68,69,70,71,72,73,74,75,76,77,78,79,80]. Furthermore, significant differences in telomerase activity levels between L-SIL versus H-SIL, L-SIL versus CC and H-SIL versus CC, with higher activity levels in the more advanced groups, were demonstrated [22,81,82]. Similarly, He et al. [69] showed significant differences in the frequency of genomic amplification of hTERC between NNL and CIN2/CIN3/SCC, between CIN1 and CIN2/CIN3/SCC, as well as between CIN2 and SCC lesions. Additionally, Chen et al. [68], further demonstrated the superiority in terms of sensitivity and specificity of hTERC and HPV DNA co-testing when compared with hTERC amplification testing alone, for cervical cancer screening: hTERC Se = 90.0% and SP = 89.6%, HPV DNA Se = 100% and Sp = 44.0%, combination hTERC and HPV DNA Se = 90.0% and Sp = 92.2% [33].

**Table 4:** hTERC up-regulation in cervical tissue biopsy specimens.

Reference, Year	Number of Biopsy Specimens	HPV Detection and Correlation with Biopsy Results	hTERC up-Regulation and Correlation with Biopsy Results
Chen et al., 2012 [68]	243, of which:	158/243, of which:	55/243, of which:
	NNL = 164	NNL = 84/164	NNL = 15/164
	CIN1 = 29	CIN1 = 24/29	CIN1 = 5/29
	CIN2 = 21	CIN2 = 21/21	CIN2 = 6/21
	CIN3 = 22	CIN3 = 22/22	CIN3 = 22/22
	CC = 7	CC = 7/7	CC = 7/7
He et al., 2012 [69]	175, of which:	N/A	86/175, of which:
	NNL = 24		NNL = 0/24
	CIN1 = 34		CIN1 = 5/34
	CIN2 = 36		CIN2 = 18/36
	CIN3 = 33		CIN3 = 23/33
	CC = 48		CC = 40/48
He et al., 2020 [70]	135, of which:	97/135	109/135
	CIN 1/2 = 65	CIN1/2 = 32/65	CIN 1/2 = 41/65
	CIN3 = 39	CIN3 = 35/39	CIN3 = 37/39
	CC = 31	CC = 30/31	CC = 31/31
Ji et al., 2019 [71]	213, of which:	103/213	64/213, of which:
	NNL = 159	75 HR, 28 LR, of which:	NNL = 29/159
	CIN1 = 31	NNL = 41 HR, 25 LR/159	CIN1 = 18/31
	CIN2 = 14	CIN1 = 16 HR, 2 LR/31	CIN2 = 9/14
	CIN3 = 7	CIN2 = 10 HR, 1 LR/14	CIN3 = 6/7
	CC = 2	CIN3 = 6 HR/7	CC = 2/2
		CC = 2 HR/2	
Jiang et al., 2010 [72]	6726, of which:	1752/2313, of which:	3250/6726, of which:
	NNL = 1257	NNL = 156/385	NNL = 124/1257
	CIN1 = 2054	CIN1 = 560/794	CIN1 = 428/2054
	CIN2 = 1387	CIN2 = 406/461	CIN2 = 952/1387
	CIN3 = 1410	CIN3 = 490/522	CIN3 = 1162/1410
	CC = 618	CC = 140/151	CC = 584/618
Jin et al., 2011 [73]	130, of which:	N/A	46/130, of which:
	NNL = 52		NNL = 2/52
	CIN1 = 33		CIN1 = 6/33
	CIN2 = 9		CIN2 = 6/9
	CIN3 = 26		CIN3 = 22/26
	CC = 10		CC = 10/10
Koeneman et al., 2019 [83]	19, of which:	19/19	15/19, of which:
	CIN2 = 3		CIN 2 = 3/3
	CIN3 = 16		CIN 3 = 12/16
Kudela et al., 2018 [74]	111, of which:	90/111, of which:	58/111, of which:
	NNL = 27	NNL = 14/27	NNL = 1/27
	CIN1 = 15	CIN1 = 7/15	CIN1 = 4/15
	CIN2 = 24	CIN2 = 24/24	CIN2 = 11/24
	CIN3 = 25	CIN3/CIS = 25/25	CIN3 = 21/25
	CC = 20	CC = 20/20	CC = 20/20
Kuglik et al., 2015 [75]	74, of which:	64/74, of which:	23/74, of which:
	NNL = 12	NNL = 10/12	NNL = 3/12
	CIN1 = 6	CIN1 = 3/6	CIN1 = 1/6
	CIN2 = 6	CIN2 = 3/6	CIN2 = 3/6
	CIN3 = 12	CIN3 = 10/12	CIN3 = 7/12
	CC = 38	CC = 34/38	CC = 33/38
Li et al., 2014 [77]	171, of which:	N/A	67/171, of which:
	NNL = 64		NNL = 6/64
	CIN1 = 26		CIN1 = 6/26
	CIN2 = 29		CIN2 = 15/29
	CIN3 = 36		CIN3 = 26/36
	CC = 16		CC = 14/16
Liu et al., 2012 [23]	114, of which:	77/114	51/114
	NNL = 27		NNL = 0/26
	CIN1 = 26		CIN1 = 4/19
	CIN2 = 16		CIN2 = 6/12
	CIN3 = 24		CIN 3 = 22/27
	CC = 21		CC = 19/19
Liu et al., 2019 [24]	150, of which:	108/150, of which:	64/150
	NNL = 32	NNL = 10/32	NNL = 4/32
	CIN1 = 38	CIN1 = 25/38	CIN1 = 13/38
	CIN2/3 = 66	CIN2/3 = 60/66	CIN2/3 = 35/66
	CC = 14	CC = 13/14	CC = 12/14
Xiang et al., 2012 [21]	92, of which:	N/A	62/92
	NNL = 20		NNL = 0/20
	CIN3 = 14		CIN3 = 12/14
	CC = 58		CC = 50/58
Yin et al., 2012 [78]	166, of which:	N/A	101/166
	NNL = 40		NNL = 0/40
	CIN1 = 27		CIN1 = 12/27
	CIN2/3 = 54		CIN2/3 = 46/54
	CC = 45		CC = 43/45
Zappacosta et al., 2015 [25]	54, of which:	52/54	20/54, of which:
	NNL = 8		NNL = 0/8
	CIN1 = 26		CIN1 = 6/26
	CIN2 = 9		CIN2 = 6/9
	CIN3 = 11		CIN3 = 8/11
Zheng et al., 2013 [79]	373, of which:	267/373, of which:	192/373, of which:
	NNL = 89	NNL = 26/89	NNL = 0/89
	CIN1 = 36	CIN1 = 19/36	CIN1 = 5/36
	CIN2 = 43	CIN2 = 32/43	CIN2 = 18/43
	CIN3 = 129	CIN3 = 119/129	CIN3 = 101/129
	CC = 76	CC = 71/76	CC = 68/76
Zhu et al., 2018 [80]	138, of which:	85/138, of which:	74/138, of which:
	NNL = 23	NNL = 4/23	NNL = 2/23
	CIN1 = 42	CIN1 = 16/42	CIN1 = 13/42
	CIN2 = 20	CIN2 = 14/20	CIN2 = 11/20
	CIN3 = 28	CIN3 = 26/28	CIN3 = 23/28
	CC = 25	CC = 25/25	CC = 25/25

**Fibronectin**

Zhou et al. [84] performed a comparative study assessing the levels of FN1 expression in 94 paired patients with CC by quantitative real-time polymerase chain reaction (qRT-PCR). They found significantly higher FN1 levels in cervical cancer tissues than in adjacent normal tissues. Furthermore, higher FN1 expression was correlated with poor prognosis.

## 4. Discussion

Currently, cervical cytology (Pap smear) is the standard screening test for CC and precancerous lesions [11]. For ASC-US and ASC-H lesions, a combination of Pap smear and HR-HPV analysis is generally recommended as a triage step before colposcopy [66]. However, these tests have low applicability: Pap smear can only identify abnormal cell morphologies and most HPV infections are self-limited, thus neither test has predictive value [22].

Despite being considered transitory, low-grade lesions, a critically large number of ASC-US and LSIL specimens had underlying CIN2 and CIN3 morphologic changes, which carry a high risk for malignant transformation [18]. Consequently, adjunctive biomarkers have been investigated in order to increase the accuracy of CC screening and to guide selection of the most appropriate treatment option.

**P16/Ki-67 staining**

p16inka (p16) is a cyclin-dependent kinase inhibitor involved in the normal cycle of somatic cells and acts as a tumor suppressor [44]. p16 overexpression is associated with keeping the retinoblastoma protein (Rbp) in an unphosphorylated state which deaccelerates cell cycle progression from G1 to S phase [85,86]. Viral oncogenes E6 and E7 are known to be drivers of proliferation, promoting and maintaining the malignant growth of cervical cells in the process of high-risk HPV-linked carcinogenesis [13,87]. p16 protein is considered a surrogate biomarker for the transforming activity of high-risk HPV and it can be detected via IHC staining of cytology or histology specimens [88,89]. p16-positivity is defined as strong and diffuse staining, meaning nuclear and/or nuclear plus cytoplasmic expression affecting the basal and para-basal cell layers and extending to the surface of the squamous epithelium on histological sections [90].

Ki-67 is a non-histone cell cycle progression antigen expressed only during the active phases of the cell cycle (G1, S, G2 and mitosis) and it is described as a biomarker for determining the growth fraction of a tumor [91]. According to IHC studies, Ki-67 is normally expressed in the basal and para-basal layers of the epithelium, whereas high-grade CIN lesions containing abnormally proliferating cells appear as increased Ki-67 staining in all layers of the squamous epithelium [19]. Isolated expression of p16 or Ki-67 within a cell may be considered physiologic, whereas simultaneous positive staining of the two biomarkers is linked with cell cycle dysregulation associated with a transformative high-risk HPV infection [13]. Co-expression of p16 and the cell cycle progression biomarker Ki-67 in one cell allows for the unequivocal identification of HPV-transformed epithelial cells and can be detected via dual immunostaining (DS) of p16/Ki-67 [92].

Additionally, p16/Ki-67 DS positivity was strongly associated with HR-HPV persistence and the presence of CIN2+ lesions [57]. One study found that p16/Ki-67 DS had sensitivity and specificity rates of 93.2% and 46.1%, respectively, for CIN3+ detection and these increased to 97.2% and 60.0% in women older than 30 years; for women with HR-HPV-positive ASC-US and LSIL, sensitivity and specificity rates were as high as 90.6% and 48.6%, respectively, which might make p16/Ki-67 DS a potent biomarker for LSIL triage [22]. Additionally, Ma et al. [36] showed that p16 immunostaining had significantly higher specificity and accuracy in predicting high-grade CIN and CC in ASC-US and LSIL specimens, as compared with HR-HPV DNA testing.

In a prospective, cross-sectional study on 599 patients, Liu et al. [65] compared the clinical performance of Pap smear, HPV DNA testing and p16/Ki-67 DS for the detection of CIN2+/VAIN2+. They found that for women who tested positive for HR-HPV and had a Pap smear ≥ ASC-US, DS reduced the number of unnecessary colposcopy referrals from 274 to 181. Additionally, DS identified four high-grade lesions that had initially negative colposcopy-guided biopsy results.

A recent meta-analysis evaluating the accuracy of p16 and p16/Ki-67 DS versus HR-HPV testing for the detection of CIN2+/CIN3+ in women with ASC-US and LSIL found that p16 staining and p16/Ki-67 DS were more specific than HR-HPV DNA testing, whereas p16 staining was less sensitive and DS has similar sensitivity [93].

Throughout the studies, however, sensitivity (Se) rates remained above 90%, whereas specificity (Sp) rates were below 50% [22], which indicates a high risk of unnecessary biopsy referrals. p16 IHC had significantly higher specificity and accuracy rates in predicting high-grade CIN and CC in ASC-US and LSIL specimens, as compared with HR-HPV DNA testing [36]. Additionally, p16 and HR-HPV co-testing had Se = 89.58% and Sp = 72.73% [33]. However, no studies analyzing the combined Se and Sp rates of cytology, HPV DNA testing and DS have been performed. On the other hand, p16 IHC was shown to be a significant negative predictor of survival [44], whereas HPV-positive CC had better survival rates [45,46,47]. Finally, according to The Lower Anogenital Squamous Terminology (LAST), p16 IHC is recommended for distinguishing between H-SIL and benign lesions mimicking precancerous lesions (immature squamous metaplasia, atrophy, repair changes and tangentially sectioned specimens) and also for the assessment of morphologically equivocal cases interpreted as L-SIL versus H-SIL [94].

Hence, given the current literature, it can be postulated that DS can be used ancillary to, or instead of HPV DNA detection, for women with ASC-US and LSIL. Additionally, p16 IHC can be used as a negative survival predictor for women with CC [44].

**Telomerase**

Telomerase is a ribonucleoprotein enzyme complex that adds 50-TTAGGG-30 repeats to the chromosomal ends known as telomeres, which play an important part in maintaining chromosomal stability during DNA replication [21,23,24]. Human telomerase consists of three subunits: one RNA component (hTERC), which functions as a template for DNA replication; one of unknown function (TP1) and the human telomerase reverse transcriptase (hTERT) [95,96]. hTERC gene expression is consistent with telomerase activity and it is generally expressed in many normal tissues [24]. However, telomerase up-regulation can reflect a malignant process as it stops cellular apoptosis, consequently leading to tumorigenesis [21,22]. The majority of studies have demonstrated the importance of increased telomerase activity as a prognostic marker in CC, its level being positively correlated with viral load, clinical stage, tumor size and lymph node metastases [96]. The activity of telomerase might be a potential method for the differential diagnosis between low-grade and high-grade precancerous cervical neoplasia, reaching Se and Sp rates of over 90% [21,23,97,98]. HR-HPV positivity and increased hTERC activity have been linked to more aggressive CC and might have an important role in future screening algorithms [23,24,25].

Furthermore, it has been suggested that hTERC amplification be used as a triage test, ancillary to HPV DNA in ASC-US and LSIL cytological samples, as a predictor of progression to more severe cervical neoplasia [21]. Studies have shown that increased telomerase activity detected by FISH analysis increased with the degree of cervical dysplasia [21]. In addition, hTERC FISH analysis significantly improved the specificity and positive predictive value of HPV DNA testing in differentiating CIN2+ from CIN2 cytological samples [25,79]. Currently, the determination of telomerase activity is not used in routine screening tests, but most authors have proposed that this method become part of future screening tests for cervical dysplasia [24,77,80,96].

Moreover, the combination of cytology, HPV DNA testing and hTERC amplification reached Se and Sp levels as high as 100% and 98.11%, respectively [68,71]. This makes hTERC an important adjunctive biomarker for CC screening and it can be recommended as an ancillary test to cytology and HPV DNA detection in women with ASC-US and LSIL lesions.

**Fibronectin**

Fibronectin is an extracellular matrix glycoprotein that plays a major role in cell differentiation, growth and migration. Furthermore, it is involved in the processes of wound healing and embryonic development, as well as oncogenic transformation. The highest levels of fibronectin expression were detected in colorectal, renal and esophageal cancers and were associated with poor prognosis [84]. Few studies have shown a significantly higher expression of fibronectin in cervical cancer tissues compared with adjacent normal tissues, but further evidence is lacking [84,99]. Consequently, the role of fibronectin as a prognostic marker in patients with CC requires additional investigation and might have potential diagnostic and therapeutic implications.

## 5. Challenges and Future Scope

CC screening and HPV vaccination campaigns are the pillars of CC prevention. However, given the financial, political and educational differences worldwide, strategies for CC prevention cannot be implemented homogenously. Access to medical care, information campaigns and health financing influence the addressability of CC screening and the treatment options. Hence, there is continuous research for more reliable and accessible biomarkers that can be used irrespective of the socioeconomic background of each country.

## 6. Conclusions

Currently, cervical cytology and HR-HPV analysis are the well-known and widely accepted screening tests for CC and precancerous lesions. However, they cannot be used to predict lesion progression to high-risk intraepithelial neoplasia. ASC-US and LSIL specimens can have underlying CIN2 and CIN3 morphologic changes, which carry a high risk for CC progression, which emphasizes the need for adjunctive biomarkers with predictive value.

p16 IHC had significantly higher specificity and accuracy rates than HPV DNA testing in predicting high-grade cervical dysplasia and CC in ASC-US and LSIL specimens. Thus, p16 IHC can be used as an alternative to HPV DNA testing in low-income countries for women with ASC-US and LSIL cytology. However, p16 and HPV DNA co-testing have better sensitivity and specificity rates (Se = 89.58% and Sp = 72.73%), which lowers the number of unnecessary colposcopy referrals, but each case should be investigated according to financial availability. Additionally, p16 can be used as a negative survival predictor for women with CC.

The combination of cytology, HPV DNA testing and hTERC FISH amplification reached sensitivity and specificity levels as high as 100% and 98.11%, respectively, which make hTERC an important, although expensive, adjunctive biomarker for CC screening. It can be recommended as an ancillary test to cytology and HPV DNA detection in women with ASC-US and LSIL lesions, in medium- and high-income countries.

## Data Availability

Not applicable.
